# Preparation of Network-Structured Carbon Nanofiber Mats Based on PAN Blends Using Electrospinning and Hot-Pressing Methods for Supercapacitor Applications

**DOI:** 10.3390/nano11092447

**Published:** 2021-09-20

**Authors:** Min-Jung Ma, Jae-Gyoung Seong, Sivaprakasam Radhakrishnan, Tae-Hoon Ko, Byoung-Suhk Kim

**Affiliations:** 1 Department of Organic Materials & Fiber Engineering, Jeonbuk National University, 567 Baekje-daero, Deokjin-gu, Jeonju-si 54896, Jeollabuk-do, Korea; mjma0829@naver.com (M.-J.M.); jaeging2@daum.net (J.-G.S.); s.rkn168@gmail.com (S.R.); 2 Department of Carbon Composites Convergence Materials Engineering, Jeonbuk National University, 567 Baekje-daero, Deokjin-gu, Jeonju-si 54896, Jeollabuk-do, Korea; 3 Department of Nano Convergence Engineering, Jeonbuk National University, 567 Baekje-daero, Deokjin-gu, Jeonju-si 54896, Jeollabuk-do, Korea

**Keywords:** carbon nanofibers, electrospinning, electrode materials, supercapacitors

## Abstract

In this work, we prepared network-structured carbon nanofibers using polyacrylonitrile blends (PAN150 and PAN85) with different molecular weights (150,000 and 85,000 g mol^−1^) as precursors through electrospinning/hot-pressing methods and stabilization/carbonization processes. The obtained PAN150/PAN85 polymer nanofibers (PNFs; PNF-73, PNF-64 and PNF-55) with different weight ratios of 70/30, 60/40 and 50/50 (*w/w*) provided good mechanical and electrochemical properties due to the formation of physically bonded network structures between the blended PAN nanofibers during the hot-processing/stabilization processes. The resulting carbonized PNFs (cPNFs; cPNF-73, cPNF-64, and cPNF-55) were utilized as anode materials for supercapacitor applications. cPNF-73 exhibited a good specific capacitance of 689 F g^−1^ at 1 A g^−1^ in a three-electrode set-up compared to cPNF-64 (588 F g^−1^ at 1 A g^−1^) and cPNF-55 (343 F g^−1^ at 1 A g^−1^). In addition, an asymmetric hybrid cPNF-73//NiCo_2_O_4_ supercapacitor device also showed a good specific capacitance of 428 F g^−1^ at 1 A g^−1^ compared to cPNF-64 (400 F g^−1^ at 1 A g^−1^) and cPNF-55 (315 F g^−1^ at 1 A g^−1^). The cPNF-73-based device showed a good energy density of 1.74 W h kg^−1^ (0.38 W kg^−1^) as well as an excellent cyclic stability (83%) even after 2000 continuous charge–discharge cycles at a current density of 2 A g^−1^.

## 1. Introduction

The ever-growing global demand for energy together with the depletion of fossil fuels makes it critical to develop sustainable and renewable energy resources [1,2,3]. High-performance electrochemical supercapacitors are acknowledged as one of the promising alternative energy storage devices due to their excellent characteristics such as high power density, rapid charge/discharge rate, and cyclic stability [4,5,6]. The development of advanced electrode materials with excellent energy storage properties is the major task for the fabrication of supercapacitor devices in real-world applications. Hence, the development of new electrode materials with improved electrochemical performance through a simple, cost-effective, and green approach is highly desirable [7,8]. So far, tremendous research efforts have been made to increase the energy density of supercapacitors without sacrificing their high power capability, rate capability, etc. Carbon nanomaterials, transition metal oxides/hydroxides, and conducting polymers are the most commonly used electrode materials for supercapacitors [8,9,10,11]. Among them, carbon-based materials are considered possible candidates for flexible energy storage applications owing to their large specific surface area, high electrical conductivity, light weight, flexibility, and fast ion response for reduced ion transport path, etc. [12,13,14]. However, at the same time, excellent mechanical stability and electrochemical properties are really tough to achieve from carbon materials. Under such demands, various approaches have been used to achieve good mechanical and electrochemical properties based on carbon materials [14,15,16,17]. Because of their poor mechanical and electrical properties, transition metal oxides/hydroxides and conducting polymers are not a proper choice as electrodes for flexible devices. Carbon nanomaterials (carbon nanofibers, graphene, and carbon nanotubes) can be the best electrode materials used for flexible devices due to their good mechanical and electrochemical properties [18,19,20,21]. They are extensively investigated for flexible devices based on graphene and carbon nanotubes because of their high conductivity and excellent mechanical stability. However, the main drawback of graphene (face-to-face aggregation of graphene sheets) and carbon nanotubes (bent, entangled, and agglomerated) is majorly reduced energy storage performance [22,23]. Carbon nanofibers are also one of the most popular methods and facile tools to achieve flexible devices for various applications due to their special properties including high porosity, large liquid permeability, good electrical conductivity, flexibility, self-standing fibrous structure, and feasibility for large-scale production [24,25,26,27,28,29]. Nevertheless, carbon nanofibers still suffer from poor mechanical properties in future applications. The improvement of mechanical properties with enhanced electrochemical properties is one of the major tasks for researchers. Here, we attempt to introduce the physical junction between the nanofibers to produce network-structured carbon nanofiber mats, leading to enhanced mechanical properties and improved electrochemical performance. Network-structured polyacrylonitrile (PAN)-based carbon nanofibers were prepared by the electrospinning and hot-pressing method. The prepared network-structured carbon nanofibers exhibit good mechanical properties, and the resulting network-structured carbon nanofibers were investigated as an anode material for energy storage applications.

## 2. Materials and Methods

### 2.1. Materials

Polyacrylonitrile blends (PAN150 and PAN85) with different molecular weights (MWs) of 85,000 and 150,000 g mol^−1^ were purchased from Sigma-Aldrich. *N*,*N*’-dimethylformamide (DMF; 99.5%) was purchased from Junsei Co. Ltd. All the reagents were of analytical grade and were used without further purification.

### 2.2. Preparation of Network-Structured Carbon Nanofibers

PAN150 and PAN85 were together dissolved in DMF under vigorous stirring at 60 °C for 12 h. The solution concentration was ca. 12 wt%, and the mass ratio of PAN150/PAN85 was controlled to be 70/30, 60/40 and 50/50 (*w/w*), respectively. Electrospinning was carried out at 10 kV with a tip-to-collector distance of 15 cm onto an Al foil with a flow rate of 0.4 mL h^−1^. The blended PAN150/PAN85 polymer nanofibers (PNFs) with different weight ratios of 70/30, 60/40 and 50/50 were designated as PNF-73, PNF-64, and PNF-55, respectively. The PNF-73, PNF-64, and PNF-55 samples were further hot-pressed (150 °C, 10 MPa) to produce the network-structured nanofiber mats by using a heating press machine (Heating Press D3P-30J, Dae Heung Science, Korea). Afterwards, it was stabilized (sPNFs) in air at 280 °C for 1 h at a heating rate of 1 °C min^−1^ and then carbonized (cPNFs) in a N_2_ atmosphere at 800 °C for 1 h at a heating rate of 5 °C min^−1^.

### 2.3. Assembly of Asymmetric Hybrid Supercapacitors

**Scheme 1:**
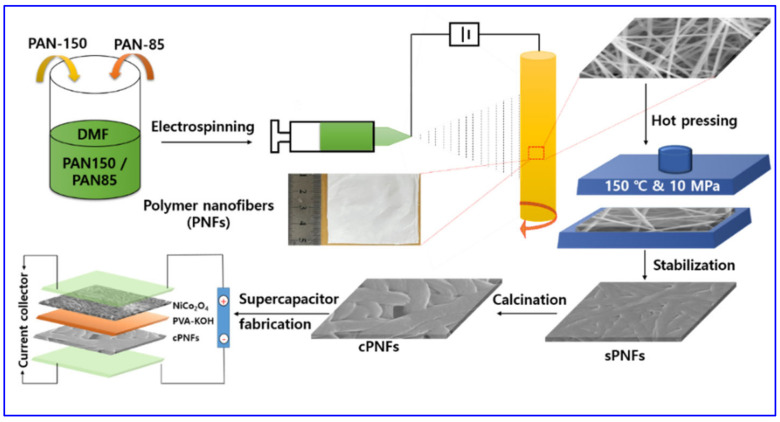
Schematic illustration for the preparation of the carbon nanofiber mats with network-structured architectures and the fabrication process of the supercapacitor device.

The solid-state asymmetric hybrid supercapacitor device was fabricated using a split test cell (EQ-STC split-able test cell, MTI Corporation, Richmond, CA, USA), where cPNF- and NiCo_2_O_4_-modified nickel foam (NiCo_2_O_4_@NF) was used as anode and cathode materials, respectively. The NiCo_2_O_4_-coated electrode was prepared according to our previous report [30]. As-prepared electrode materials were assembled by sandwiching with the PVA-KOH gel electrolyte. The PVA-KOH gel electrolyte was prepared by adding 2 g of PVA into 20 mL 2 M KOH solution under stirring until the solution became clear [31]. The working area of asymmetric supercapacitor electrode materials was controlled to be 1 cm × 1 cm. The mass loading of cathode and anode materials was about 2 mg and 2 mg, respectively. The assembled device was dried at room temperature to remove the excess water from the gel, and finally the prepared device was placed inside the plastic tube for protecting the device from physical damage. Scheme 1 depicts the detailed schematic outline for the preparation of the asymmetric cPNFs//NiCo_2_O_4_ supercapacitor devices (Scheme 1).

### 2.4. Electrochemical Characterization

Electrochemical measurements were carried out under a three-electrode set-up such as cyclic voltammetry (CV) and galvanostatic charge/discharge (GCD). In a three-electrode set-up, the prepared samples, platinum foil, and Ag/AgCl were used as the working, counter, and reference electrode, respectively. Electrochemical impedance spectroscopy (EIS) measurements were carried out in the frequency range of 100 kHz to 1 Hz at an applied amplitude of 0.01 V. 3M KOH was used as electrolyte for all the electrochemical experiments.

### 2.5. Characterization

A field-emission scanning electron microscope (FE-SEM; JEOL JSM-5900) was used to analyze the surface morphologies. The phase purity and crystalline phase of the samples were characterized using X-ray diffraction (XRD) patterns through a Rigaku X-ray diffractometer with Cu-K_a_ radiation from 10° to 80°. The thermal behavior was investigated by differential scanning calorimetry (DSC; DSC Q20; Waters, Milford, MA, USA) operating from room temperature to 320 °C under the air atmosphere. Mechanical properties were performed by using a universal testing machine (AG-5000G; Shimadzu Co., Kyoto, Japan) under a cross-head speed of 5 mm min^−1^ at room temperature. In accordance with ASTM D-638, the samples were prepared in the shape of a dumbbell, and then tensile tests were conducted on at least 3~5 specimens and the average values were reported.

## 3. Results and Discussion

**Figure 1:**
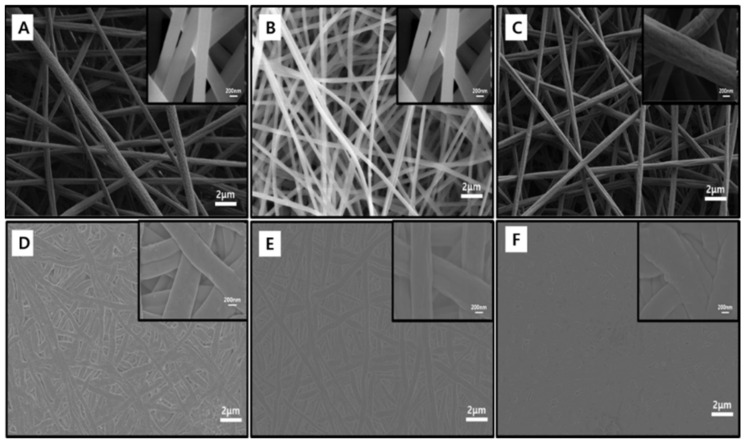
FE-SEM images of as-spun nanofibers ((**A**) PNF-73, (**B**) PNF-64, and (**C**) PNF-55) and hot-pressed nanofibers ((**D**) PNF-73, (**E**) PNF-64, and (**F**) PNF-55) produced from blended PAN solutions. The inset shows the corresponding highly magnified FE-SEM images.

**Figure 2:**
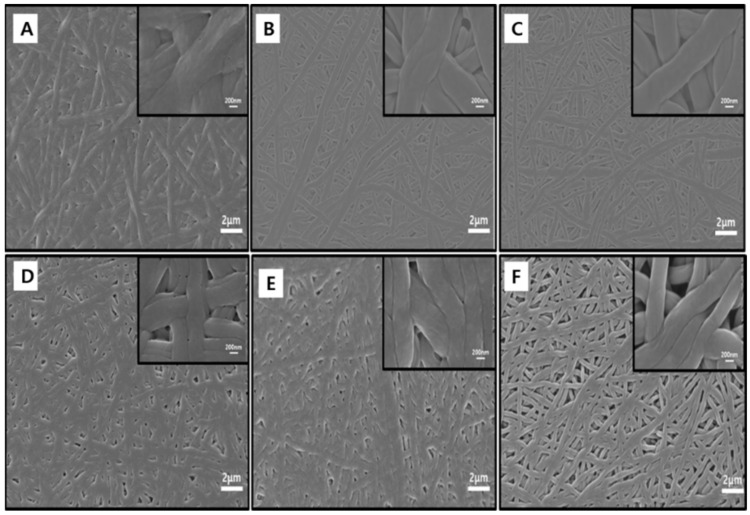
FE-SEM images of the stabilized ((**A**) sPNF-73, (**B**) sPNF-64, and (**C**) sPNF-55) and carbonized ((**D**) cPNF-73, (**E**) cPNF-64, and (**F**) cPNF-55) hot-pressed nanofibers. The inset shows the corresponding highly magnified FE-SEM images.

The blended PANs composed of two different MWs with different weight ratios (PAN150/PAN85 = 70/30, 60/40, 50/50, *w/w*) were used as precursors to prepare carbon nanofiber mats with network-structured architectures. Figure 1 shows the FE-SEM images of electrospun PAN-based nanofibers (A: PNF-73, B: PNF-64, and C: PNF-55) produced from the blended PAN150/PAN85 solutions. The average diameters of as-spun PNF-73, PNF-64, and PNF-55 were measured to be 472 ± 17, 371 ± 5, and 455 ± 11 nm, respectively. After hot-press treatment (at 150 °C under 10 MPa), the hot-pressed PNFs exhibited smooth and networked-structured morphologies (Figure 1D–F), compared to as-spun PNFs. As seen in Figure 2 (D: PNF-73, E: PNF-64, and F: PNF-55), the average diameters of hot-pressed PNF-73, PNF-64, and PNF-55 became larger and were found to be 623 ± 8, 624 ± 4, and 579 ± 5 nm, respectively. The mechanical properties of as-prepared polymer nanofibers (PNFs) were also investigated (Figure S1). Firstly, the tensile strengths (PNF-55: ~12.8 MPa, PNF-64: ~7.0 MPa, and PNF-73: ~10.2 MPa) of the hot-pressed PNFs were higher than those of pure PAN85 (~4.5 MPa) and PAN150 (~5.1 MPa) (Figure S2), confirming that the mixing effects of PAN150/PAN85 with different MWs can provide the enhanced mechanical properties of the hot-pressed PNFs, due to the formation of network-structured architectures. Furthermore, PNF-55 exhibited superior tensile strength (~12.8 MPa) and elongation at break (~61.8%) than PNF-64 and PNF-73. Figure 2 shows FE-SEM images of the stabilized (A: sPNF-73, B: sPNF-64, and C: sPNF-55) and carbonized (D: cPNF-73, E: cPNF-64, and F: cPNF-55) hot-pressed nanofibers. After the stabilization and carbonization processes, the network-structured fibrous morphologies were retained, as seen in Figure 2. In addition, the fiber diameters of the sPNFs were not largely changed, while their diameter was significantly decreased after the carbonization treatment at 800 °C, due to the decomposition of non-carbon atoms [32]. The average diameters of the carbonized (D: cPNF-73, E: cPNF-64, F: cPNF-55) hot-pressed nanofibers were measured to be 505 ± 17, 470 ± 20, and 419 ± 3 nm, respectively. Importantly, it was concluded that the fibrous morphologies and membrane nature with the pores were clearly maintained.

**Figure 3:**
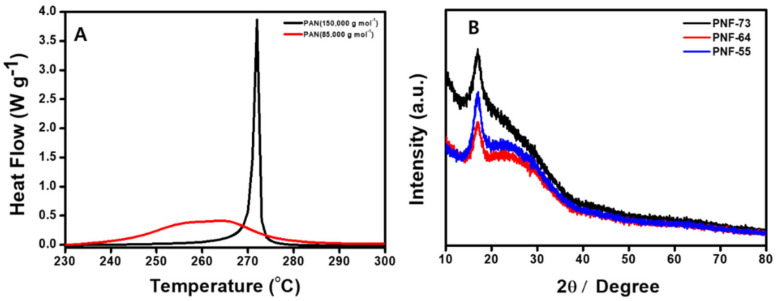
(**A**) DSC curves of as-spun PANs (PAN150 and PAN85) with two different MWs. (**B**) XRD patterns of the carbonized (cPNF-73, cPNF-64, and cPNF-55) hot-pressed nanofibers.

Figure 3A shows the DSC thermograms of the as-spun PANs (PAN85 and PAN150) under a nitrogen atmosphere. The results clearly showed that PAN150 had a strong, intense peak, whereas PAN85 showed a broad exothermic peak. The highly intense exothermic peak of PAN150 is due to the cyclization process initiated through the free radical mechanism [33]. Moreover, the lower curing temperature of PAN85 could be beneficial for achieving the network-structured morphologies in the blended PAN150/PAN85 nanofibers during the stabilization step [34]. Figure 3B shows the XRD patterns of the carbonized (cPNF-73, cPNF-64, and cPNF-55) hot-pressed nanofibers. A similar XRD pattern was observed for all the samples, and the observed peak at approximately 2θ = 17° was correlated to the (100) plane of the pseudo-hexagonal lattice of the C≡N groups [35], confirming its good crystalline nature.

**Figure 4:**
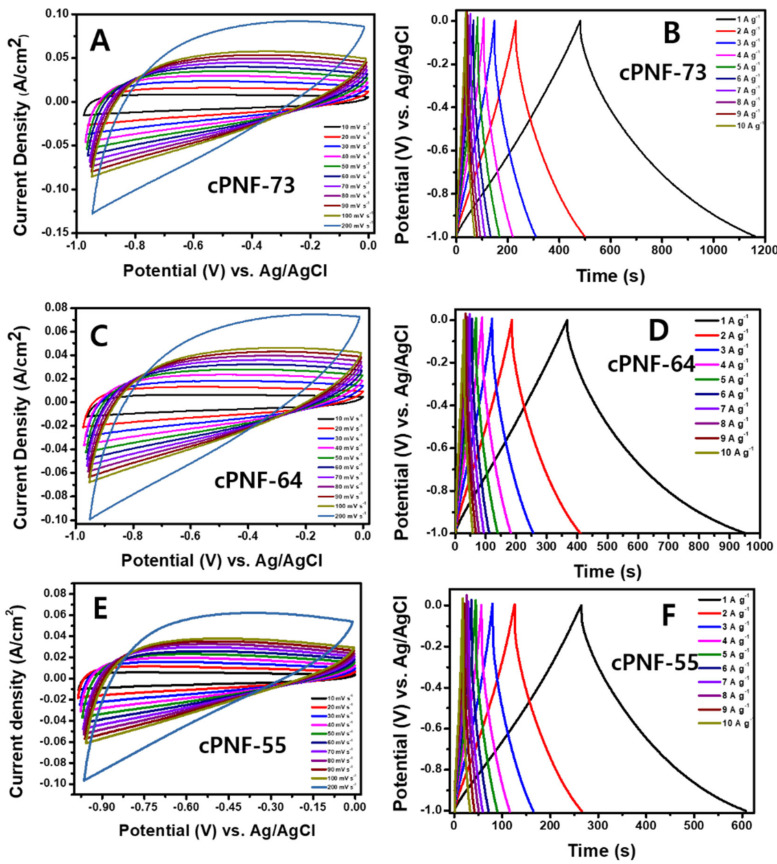
CV curves of (**A**) cPNF-73, (**C**) cPNF-64, and (**E**) cPNF-55 electrodes in 3 M KOH at scan rates varying from 10 to 200 mV s^−1^. The GCD test of (**B**) cPNF-73, (**D**) cPNF-64, and (**F**) cPNF-55 electrodes at current densities varying from 1 to 10 A g^−1^.

The obtained network-structured carbon nanofiber mats (cPNFs: cPNF-73, cPNF-64, and cPNF-55) were further used for the electrochemical studies. Figure 4 shows the CV and GCD tests of cPNF-modified electrodes in 3 M KOH. All the CV curves exhibited good rectangle behavior at various scan rates. cPNFs-73 (Figure 4A) showed the higher peak current density (90 mA cm^−2^) compared to cPNFs-64 (Figure 4C; 70 mA cm^−2^) and cPNFs-55 (Figure 4E; 60 mA cm^−2^). These preliminary CV studies clearly implied that cPNFs-based electrodes had excellent electrochemical double-layer capacitance (EDLC) properties and therefore could be useful as anode materials for electrochemical supercapacitor applications. To verify the effect of the hot-pressing method, we prepared cPNF-73, cPNF-64, and cPNF-55 without the hot-pressing process under identical conditions. The obtained carbon nanofibers were further investigated by CV at different scan rates from 10 to 200 mV s^−1^ in 3 M KOH (Figure S3). The obtained CV profile was similar to that of hot-pressed cPNFs, while the current density of hot-pressed cPNFs was higher than that of the PNFs that were not hot-pressed, confirming that the network structures formed in carbon nanofiber mats clearly improved the electrochemical performance. Hence, the hot-pressed cPNFs were further used for detailed investigations.

**Figure 5:**
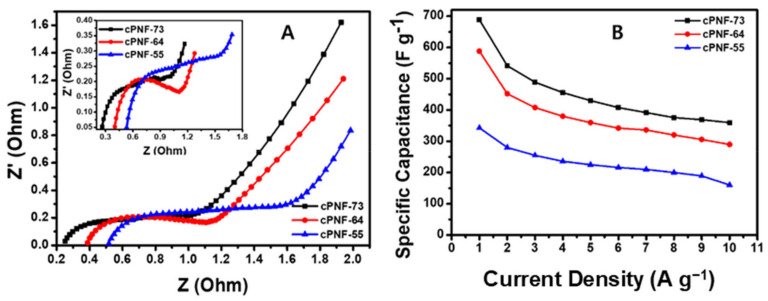
(**A**) ElS of the cPNF-73, cPNF-64, and cPNF-55 electrodes. (**B**) Specific capacitance vs. current density plots of cPNF-73, cPNF-64, and cPNF-55.

The fabricated cPNF-based electrodes were further investigated for the GCD test at various current densities (from 1 A to 10 A g^−1^), as shown in Figure 4B,D,F. All the GCD curves (cPNF-73, cPNF-64, and cPNF-55) showed symmetric charge–discharge behavior without any pseudocapacitive behavior, verifying that the cPNF-based electrodes undergo the EDLC mechanism. The specific capacitance values of cPNF-based electrodes were calculated using Equation (1) at current densities varying from 1 A g^−1^ to 10 A g^−1^. The maximum specific capacitance was observed for the cPNF-73 electrode (689 F g^−1^ @ 1 A g^−1^), which was higher than that of cPNF-64 (588 F g^−1^ @ 1 A g^−1^) and cPNF-55 (343 F g^−1^ @ 1 A g^−1^). Furthermore, on increasing the applied current density from 1 A g^−1^ to 10 A g^−1^, the specific capacitance values were gradually decreased from 689 to 360 for cPNF-73, 588 to 290 for cPNF-64, and 343 to 160 F g^−1^ for cPNF-55, respectively (Figure 5B). The GCD results clearly showed that the cPNF-73 electrode had a better rate capability (52%) than cPNF-64 (49%) and cPNF-55 (46%).

Further, the charge transfer resistance of the fabricated electrodes was studied by EIS (Figure 5A). The Nyquist plot showed that the cPNF-based electrode materials had a lower charge transfer resistance value (<1 Ω), suggesting the superior electrical conductivity of cPNF-based electrodes due to good electrical contact between the cPNF-based electrode and the current collector. As a result, the cPNF-73 electrode had a smaller semi-circle in the higher frequency regions, indicating less resistance compared to other cPNF-based electrodes. The specific capacitance of the electrode was derived using the following equation:

Cs = (I × t)/(m × ΔV)(1)

where I is the discharge current (A), t is the discharge time (s), V is the potential window (V) of the electrode, Cs is the specific capacitance (F g^−1^) of the electrode, and m is the mass (g) of the active material in the electrode.

**Figure 6:**
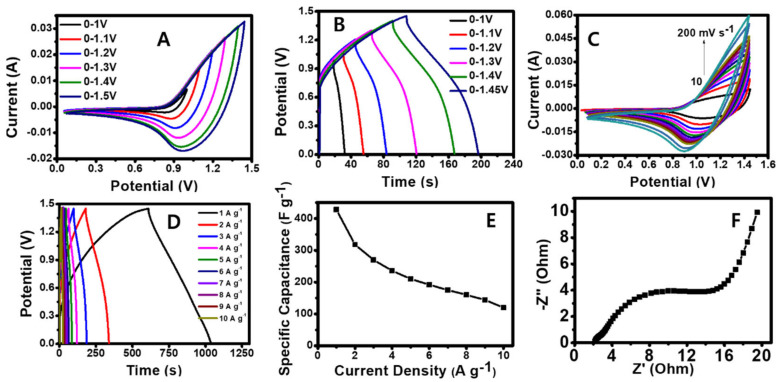
(**A**) CV curves of the cPNF-73 hybrid supercapacitor at different potential windows at a fixed scan rate of 50 mV s^−1^. (**B**) GCD curves of the cPNF-73 hybrid device at various voltage ranges. (**C**) CV curves of the supercapacitor cPNF-73 device at scan rates varying from 10 to 200 mV s^−1^. (**D**) GCD curves of the cPNF-73 hybrid device at different current densities from 1 to 10 A g^−1^. (**E**) Plot of specific capacitance vs. current density. (**F**) EIS of the cPNF-73 hybrid device.

**Figure 7:**
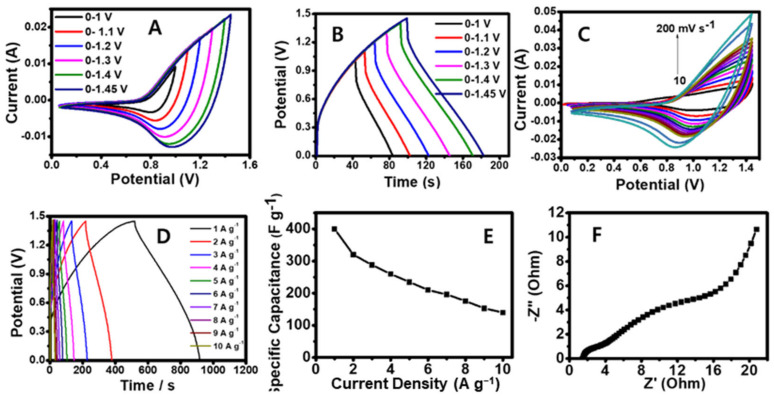
(**A**) CV curves of the cPNF-64 hybrid supercapacitor at different potential windows at a fixed scan rate of 50 mV s^−1^. (**B**) GCD curves of the cPNF-64 hybrid device at various voltage ranges. (**C**) CV curves of the supercapacitor cPNF-64 device at scan rates varying from 10 to 200 mV s^−1^. (**D**) GCD curves of the cPNF-64 hybrid device at different current densities from 1 to 10 A g^−1^. (**E**) Plot of specific capacitance vs. current density. (**F**) EIS of the cPNF-64 hybrid device.

**Figure 8:**
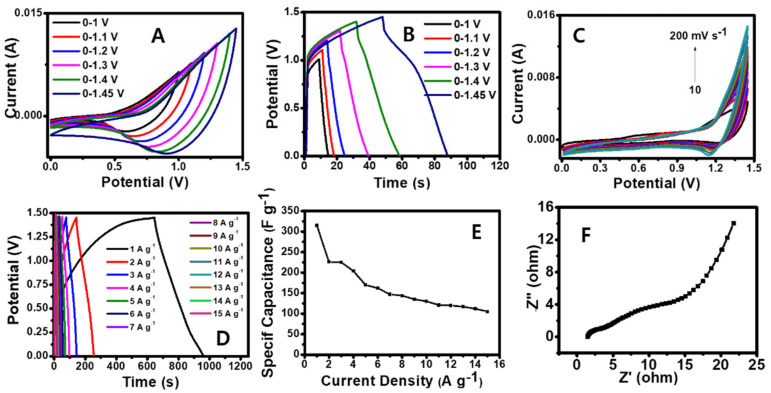
(**A**) CV curves of the cPNF-55 hybrid supercapacitor at different potential windows at a fixed scan rate of 50 mV s^−1^. (**B**) GCD curves of the cPNF-55 hybrid device at various voltage ranges. (**C**) CV curves of the supercapacitor cPNF-55 device at scan rates varying from 10 to 200 mV s^−1^. (**D**) GCD curves of the cPNF-55 hybrid device at different current densities from 1 to 10 A g^−1^. (**E**) Plot of specific capacitance vs. current density. (**F**) EIS of the cPNF-55 hybrid device.

The hybrid asymmetric supercapacitor was designed using as-prepared cPNF-73, cPNF-64, and cPNF-55 electrodes and NiCo_2_O_4_ as negative and positive electrodes, respectively. PVA-KOH gel was used as a separator to fabricate the hybrid supercapacitor device. The fabricated hybrid device was tested with various electrochemical characterizations to investigate the specific capacitance, cycle life, rate capability, and charge transfer resistance (Figure 6, Figure 7 and Figure 8). To optimize the working potential window of the fabricated cPNFs //NiCo_2_O_4_ supercapacitor device, CV (Figure 6A, Figure 7A and Figure 8A) and GCD (Figure 6B, Figure 7B and Figure 8B) measurements were recorded for the cPNF-73-, cPNF-64- and cPNF-55-based hybrid devices with various potential windows. The potential optimization study suggested that the device had a large potential window up to 1.45 V. Further, the CV test of cPNF-73 (Figure 6C), cPNF-64 (Figure 7C), and cPNF-55 (Figure 8C) electrodes was performed at scan rates varying from 10 to 200 mV s^−1^. The current density of the devices linearly increased with respect to the scan rates, suggesting the good performance of the device. As a result, the cPNF-73-based hybrid device exhibited higher current density (56 mA cm^−2^ measured at 1.4 V) compared to cPNF-64 (46 mA cm^−2^) and cPNF-55 (12 mA cm^−2^)-based devices. The GCD test was also recorded for the fabricated cPNF-73 (Figure 6D), cPNF-64 (Figure 7D), and cPNF-55 (Figure 8D) hybrid devices in the potential window between 0 and 1.45 V at applied current densities varying from 1 to 10 A g^−1^. The estimated specific capacitance values versus current density of the fabricated cPNF-73 (Figure 6E), cPNF-64 (Figure 7E), and cPNF-55 (Figure 8E) devices were recorded. The cPNF-73-based supercapacitor device delivered a maximum specific capacitance of 428 F g^−1^ at 1 A g^−1^, which was higher than that of the cPNF-64 (400 F g^−1^) and cPNF-55 (315 F g^−1^)-based devices at an applied current density of 1 A g^−1^. In addition, the specific capacitance values of the cPNFs-based devices decreased with increasing current density because the thickness of the electrical double layer decreased with increasing the charging current [36]. The capacitance retention was calculated for the fabricated cPNF-based device at a current density of 10 A g^−1^. cPNF-55 (41%) exhibited higher capacitance retention than those of cPNF-73 (28%) and cPNF-64 (35%). Further, the interfacial properties of the fabricated cPNF-73 (Figure 5F), cPNF-64 (Figure 6F), and cPNF-55 (Figure 7F) hybrid devices were evaluated using EIS. The values of the charge transfer resistance (R_CT_) were evaluated as 12, 14, and 17 Ω for cPNF-73, cPNF-64, and cPNF-55. The lower R_CT_ values suggested that the fabricated cPNF-73-based hybrid devices had good electrical contact between the electrode materials and the substrate. The cyclic stability is an important parameter of the supercapacitor device for real usage. Hence, the cyclic stability test was investigated by using continuous GCD tests. It was observed that the cycle test showed excellent cycle life performances of around 80%, 80%, and 79% for cPNF-73, cPNF-64, and cPNF-55, respectively, even after 2000 cycles (Figure S4). These electrochemical results clearly demonstrated that the fabricated cPNF-73-based hybrid device could be more suitable for real-time applications.

The energy density (E) and power density (P) of the assembled cPNFs//NiCo_2_O_4_ supercapacitor device were calculated based on the following equations:

E = 1/8C_sp_ΔV^2^ (2)

**Figure 9:**
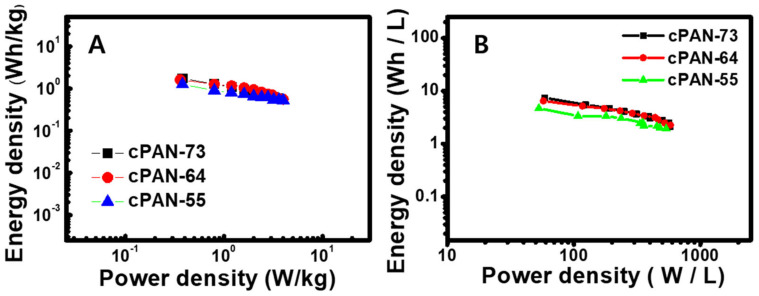
(**A**) Gravimetric and (**B**) volumetric energy and power densities of the cPAN-73, cPAN-64, and cPAN-55 hybrid devices.

P = E/Δt

where C_sp_ is the specific capacitance (F g^−1^), Δt is the galvanostatic discharge time (s) and ΔV is the potential window (V) of the device. Figure 9 shows the gravimetric and volumetric Ragone plots of the cPNFs//NiCo_2_O_4_ supercapacitor device. The cPNF-73 hybrid device demonstrated a good gravimetric energy density of 1.74 W h kg^−1^ for a specific power of 0.38 kW kg^−1^. In addition, the volumetric energy density of cPNF-73 delivered a maximum of 7.44 Wh L^−1^ at a power density of 58.8 W L^−1^. The obtained power and energy density values were very much comparable with literature reports [37,38,39] (Table S1).

## 4. Conclusions

The network-structured carbon nanofibers, which were produced by the electrospinning and hot-pressing method, were used as anode materials for supercapacitor applications. The obtained PAN150/PAN85 polymer nanofibers (PNFs; PNF-73, PNF-64, and PNF-55) with different weight ratios of 70/30, 60/40, and 50/50 (*w/w*) showed good mechanical and electrochemical properties due to the formation of physically bonded network structures between the blended PAN nanofibers during the hot-processing/stabilization process. The obtained cPNF-73 delivered a good specific capacitance of 689 F g^−1^ at 1 A g^−1^ compared to the cPNF-64 (588 F g^−1^ at 1 A g^−1^) and cPNF-55 (343 F g^−1^ at 1 A g^−1^). Furthermore, an asymmetric hybrid cPNF-73//NiCo_2_O_4_ supercapacitor device exhibited a good specific capacitance of 428 F g^−1^ at 1 A g^−1^ compared to cPNF-64 (400 F g^−1^ at 1 A g^−1^) and cPNF-55 (315 F g^−1^ at 1 A g^−1^). The fabricated cPNF-73-based device showed an excellent cyclic stability (83%) even after 2000 continuous charge–discharge cycles at a current density of 2 A g^−1^, and also capacity retention (28%) at a current density of 10 A g^−1^. The assembled cPNF-73//NiCo_2_O_4_ delivered a good gravimetric energy density of 1.74 W h kg^−1^ for a specific power density of 0.38 kW kg^−1^ and a volumetric energy density of 7.44 Wh L^−1^ at a power density of 58.8 W L^−1^. This approach will create a great opportunity to prepare various networked-structured polymer nanofibers with attractive properties for different applications.

## Data Availability

All data are available upon reasonable request.

## Supplementary Materials

The following are available online at https://www.mdpi.com/article/10.3390/nano11092447/s1: Figure S1: Stress–strain curves of the hot-pressed PAN and blended PAN150/PAN85 (PNF-73, PNF-64, and PNF-55) nanofibers; Figure S2: Stress–strain curves of as-spun PAN150 and PAN85 nanofibers; Figure S3: CV curves of cPNFs (cPNF-73, cPNF-64, and cPNF-55) electrodes with and without the hot-pressing method in 3 M KOH; Figure S4: Cyclic stability of the fabricated cPNF devices (A: cPNF-73, B: cPNF-74, and C: cPNF-55) at 2 A g^−1^; Table S1: Electrochemical performances of various hybrid supercapacitors.

## References

[1] Jorn-am T., Praneerad J., Attajak R., Sirisit N., Manyam J., Paoprasert P. (2021). Quasi-solid, bio-renewable supercapacitor with high specific capacitance and energy density based on rice electrolytes and rice straw-derived carbon dots as novel electrolyte additives. Colloids Surf. A: Physicochem. Eng. Asp..

[2] Kong M., Wang Z., Wang W., Min M., Liu D., Hao S., Kong R., Du G., Asiri A.M., Yao Y. (2017). NiCoP nanoaray: A superior pseudocapacitor electrode with high areal capacitance. Chem. A Eur. J..

[3] Ko T.H., Radhakrishnan S., Seo M.K., Khil M.S., Kim H.Y., Kim B.S. (2017). A green and scalable dry synthesis of NiCo_2_O_4_/graphene nanohybrids for high-performance supercapacitor and enzymeless glucose biosensor applications. J. Alloys Compd..

[4] Li Y., Zhu G., Huang H., Xu M., Lu T., Pan L. (2019). A N, S dual doping strategy via electrospinning to prepare hierarchically porous carbon polyhedral embedded carbon nanofibers for flexible supercapacitors. J. Mater. Chem. A.

[5] Saravanakumar B., Ko T.H., Kim B.S. (2018). Rational design of binder-free ZnCo_2_O_4_ and Fe_2_O_3_ decorated porous 3D Ni as high-performance electrodes for asymmetric supercapacitor. Ceram. Int..

[6] Chen C., Zhao M., Cai Y., Zhao G., Xie Y., Zhang L., Zhu G., Pan L. (2021). Scalable synthesis of strutted nitrogen doped hierarchical porous carbon nanosheets for supercapacitors with both high gravimetric and volumetric performances. Carbon.

[7] Wannasen L., Mongkolthanaruk W., Swatsitang E., Pavasant P., Pinitsoontorn S. (2021). Co_2_P_2_O_7_ Microplate/Bacterial Cellulose–Derived Carbon Nanofiber Composites with Enhanced Electrochemical Performance. Nanomaterials.

[8] Liang R., Du Y., Xiao P., Cheng J., Yuan S., Chen Y., Chen J. (2021). Transition Metal Oxide Electrode Materials for Supercapacitors: A Review of Recent Developments. Nanomaterials.

[9] Lei D., Li X.D., Seo M.K., Khil M.S., Kim H.Y., Kim B.S. (2017). NiCo_2_O_4_ nanostructure-decorated PAN/lignin based carbon nanofiber electrodes with excellent cyclability for flexible hybrid supercapacitors. Polymer.

[10] Tian L., Mingxian L., Dazhang Z., Gan L., Chen T. (2018). Nanocarbon-based materials for flexible all-solid-state supercapacitors. Adv. Mater..

[11] Cao Y., Liang J., Li X., Yue L., Liu Q., Lu S., Abdullah M.A., Jianming H., Luo Y., Sun X. (2021). Recent advances in perovskite oxides as electrode materials for supercapacitors. Chem. Commun..

[12] Chen L., Xu X., Wan L., Zhu G., Li Y., Lu T., Albaqami M.D., Pan L., Yamauchi Y. (2021). Carbon-incorporated Fe_3_O_4_ nanoflakes: High-performance faradic materials for hybrid capacitive deionization and supercapacitors. Mater. Chem. Front..

[13] Liang J., Zhao H., Yue L., Fan G., Li T., Lu S., Sun X. (2020). Recent advances in electrospun nanofibers for supercapacitors. J. Mater. Chem. A.

[14] Xu X., Liu Y., Wang M., Zhu C., Lu T., Zhao R., Pan L. (2016). Hierarchical hybrids with microporous carbon spheres decorated three-dimensional graphene frameworks for capacitive applications in supercapacitor and deionization. Electrochim. Acta.

[15] Pan Z., Liu M., Yang J., Qiu Y., Li W., Xu Y., Zhang Y. (2017). High electroactive material loading on a carbon nanotube@ 3D graphene aerogel for high-performance flexible all-solid-state asymmetric supercapacitors. Adv. Funct. Mater..

[16] Zhang Z., Wang L., Li Y., Wang Y., Zhang J., Guan G., Peng H. (2017). Nitrogen-doped core-sheath carbon nanotube array for highly stretchable supercapacitor. Adv. Energy Mater..

[17] Xiao Y., Xu Y., Zhang K., Tang X., Huang J., Yuan K., Chen Y. (2020). Coaxial electrospun free-standing and mechanically stable hierarchical porous carbon nanofiber membranes for flexible supercapacitors. Carbon.

[18] Chen W., Wang H., Lan W., Li D., Zhang A., Liu C. (2021). Construction of sugarcane bagasse-derived porous and flexible carbon nanofibers by electrospinning for supercapacitors. Ind. Crop. Prod..

[19] Mytafides C.K., Tzounis L., Karalis G., Formanek P., Paipetis A.S. (2021). Fully printed and flexible carbon nanotube-based thermoelectric generator capable for high-temperature applications. J. Power Sources.

[20] Zhang Y., Hui S., Lin X., Ying Z., Li Y., Xie J. (2021). Novel effective strategy for high-performance biomass-based super-flexible hierarchically porous carbon fibrous film electrode for supercapacitors. J. Alloys Compd..

[21] Tao Y., Liu W., Li Z., Zheng Y., Zhu X., Wang H., Chen H. (2021). Boosting supercapacitive performance of flexible carbon via surface engineering. J. Colloid Interface Sci..

[22] Lee S.W., Yabuuchi N., Gallant B.M., Chen S., Kim B.S., Hammond P.T., Shao-Horn Y. (2010). High-power lithium batteries from functionalized carbon-nanotube electrodes. Nat. Nanotechnol..

[23] Xiong G., He P., Lyu Z., Chen T., Huang B., Chen L., Fisher T.S. (2018). Bioinspired leaves-on-branchlet hybrid carbon nanostructure for supercapacitors. Nat. Commun..

[24] Liu Y., Hao M., Chen Z., Liu L., Liu Y., Yang W., Ramakrishna S. (2020). A review on recent advances in application of electrospun nanofiber materials as biosensors. Curr. Opin. Biomed. Eng..

[25] Alimohammadi M., Aghli Y., Fakhraei O., Moradi A., Passandideh-Fard M., Ebrahimzadeh M.H., Mousavi Shaegh S.A. (2020). Electrospun nanofibrous membranes for preventing tendon adhesion. ACS Biomater. Sci. Eng..

[26] Zhang Z., Wu X., Kou Z., Song N., Nie G., Wang C., Verpoort F., Mu S. (2022). Rational Design of Electrospun Nanofiber-Typed Electrocatalysts for Water Splitting: A Review. Chem. Eng. J..

[27] Forghani S., Almasi H., Moradi M. (2021). Electrospun nanofibers as food freshness and time-temperature indicators: A new approach in food intelligent packaging. Innov. Food Sci. Emerg. Technol..

[28] Guo H., Chen Y., Li Y., Zhou W., Xu W., Pang L., Jiang S. (2021). Electrospun fibrous materials and their applications for electromagnetic interference shielding: A review. Compos. Part. A Appl. Sci. Manuf..

[29] Cui C., Sun S., Wu S., Chen S., Ma J., Zhou F. (2021). Electrospun chitosan nanofibers for wound healing application. Eng. Regen..

[30] Park J., Ko T.H., Balasubramaniam S., Seo M.K., Khil M.S., Kim H.Y., Kim B.S. (2019). Enhancing the performance and stability of NiCo_2_O_4_ nanoneedle coated on Ni foam electrodes with Ni seed layer for supercapacitor applications. Ceram. Int..

[31] Ranjith K.S., Kwak C.H., Hwang J.U., Ghoreishian S.M., Raju G.S.R., Huh Y.S., Im J.S., Han Y.K. (2020). High-performance all-solid-state hybrid supercapacitors based on surface-embedded bimetallic oxide nanograins loaded onto carbon nanofiber and activated carbon. Electrochim. Acta.

[32] Hajir Bahrami S., Bajaj P., Sen K. (2003). Thermal behavior of acrylonitrile carboxylic acid copolymers. J. Appl. Polym. Sci..

[33] Fitzer E., Frohs W., Heine M. (1986). Optimization of stabilization and carbonization treatment of PAN fibers and structural characterization of the resulting carbon fibers. Carbon.

[34] Yu X., Ma M., Jan J. (2018). Preparation of multi-layer nylon-6 nanofiber membranes by electrospinning and hot pressing method for dye filtration. RSC Adv..

[35] Shen T., Li C., Haley B., Desai S., Strachan A. (2018). Crystalline and pseudo-crystalline phases of polyacrylonitrile from molecular dynamics: Implications for carbon fiber precursors. Polymer.

[36] Ratajczak P., Suss M.E., Kaasik F., Beguin F. (2019). Carbon electrodes for capacitive technologies. Energy Storage Mater..

[37] Cottineau T., Toupin M., Delahaye T., Brousse T., Belanger D. (2006). Nanostructured transition metal oxides for aqueous hybrid electrochemical supercapacitors. Appl. Phys. A.

[38] Raj C.S., Manikandan R., Rajesh M., Sivakumar P., Jung H., Das S.J., Kim B.C. (2021). Cornhusk mesoporous activated carbon electrodes and seawater electrolytes: The sustainable sources for assembling retainable supercapacitor module. J. Power Sources.

[39] Madani S., Falamaki C., Alimadadi H., Aboutalebi S.H. (2019). Binder-free reduced graphene oxide 3D structures based on ultra large graphene oxide sheets: High performance green micro-supercapacitor using NaCl electrolyte. J. Energy Storage.

