# Supplementary material for: Preparation of Network-Structured Carbon Nanofiber Mats Based on PAN Blends Using Electrospinning and Hot-Pressing Methods for Supercapacitor Applications
Source: Nanomaterials (Basel). 2021 Sep 20;11(9):2447. doi: 10.3390/nano11092447 (PMC8467548; doi:10.3390/nano11092447)
Supplement: Supplementary file 1 [file nanomaterials-11-02447-s001.zip › nanomaterials-1386031-supplementary.pdf]

SUPPORTING INFORMATION

Preparation of mechanically enhanced carbon nanofiber mats based on PAN blends using electrospinning and hot pressing methods for supercapacitor application

Min-Jung Ma [1,2], Jae-Gyoung Seong [1,2], Sivaprakasam Radhakrishnan [1], Tae-Hoon Ko [3,*], Byoung-Suhk Kim [1,2,*]

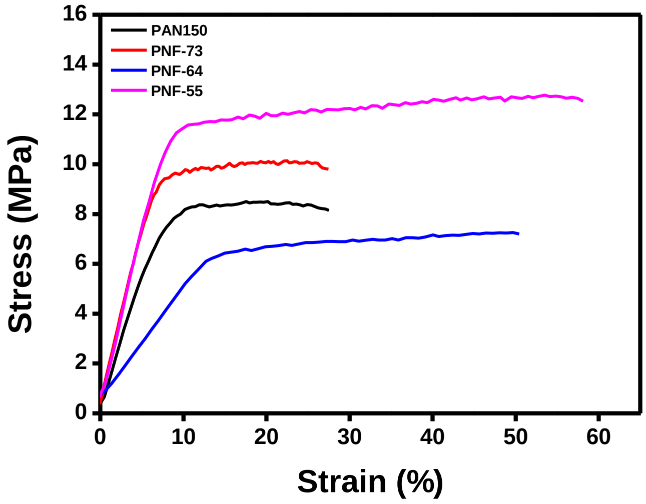

Figure S1. Stress-strain curves of the hot-pressed PAN and blended PAN150/PAN85 (PNF-73, PNF-64, and PNF-55) nanofibers.

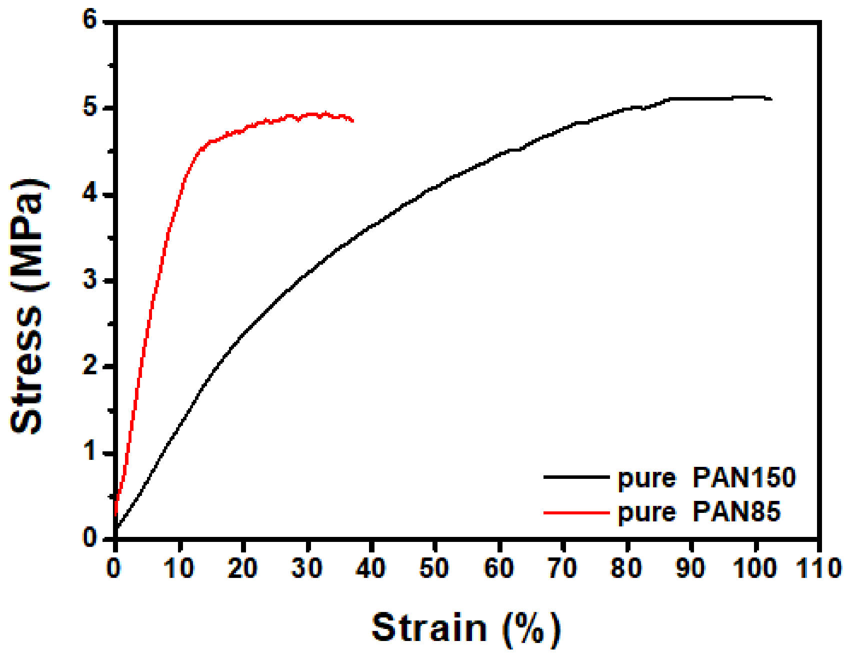

Figure S2. Stress-strain curves of as-spun pure PAN150 and PAN85 nanofibers

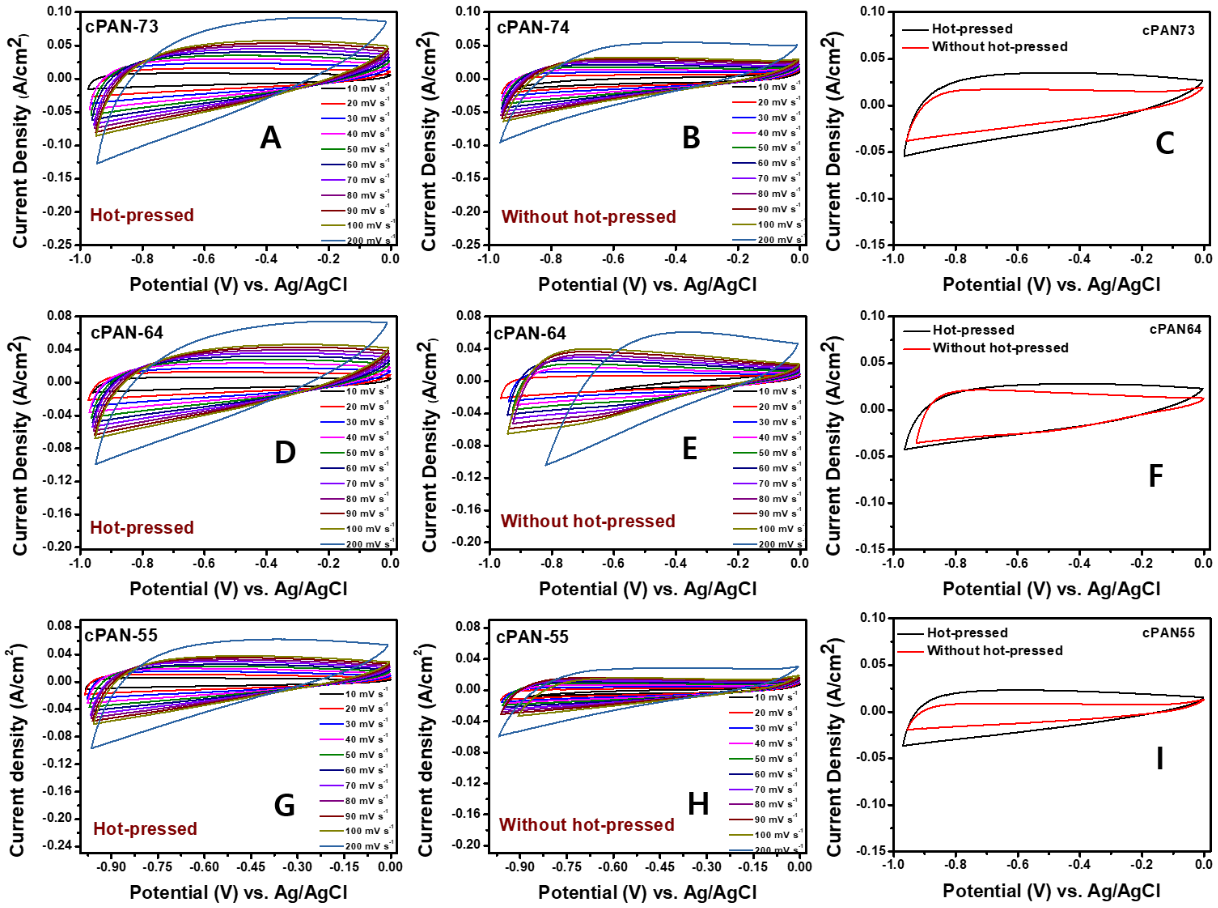

Figure S3. CV curves of cPNFs (cPNF-73, cPNF-64 and cPNF-55) with and without hot-pressed method in 3M KOH.

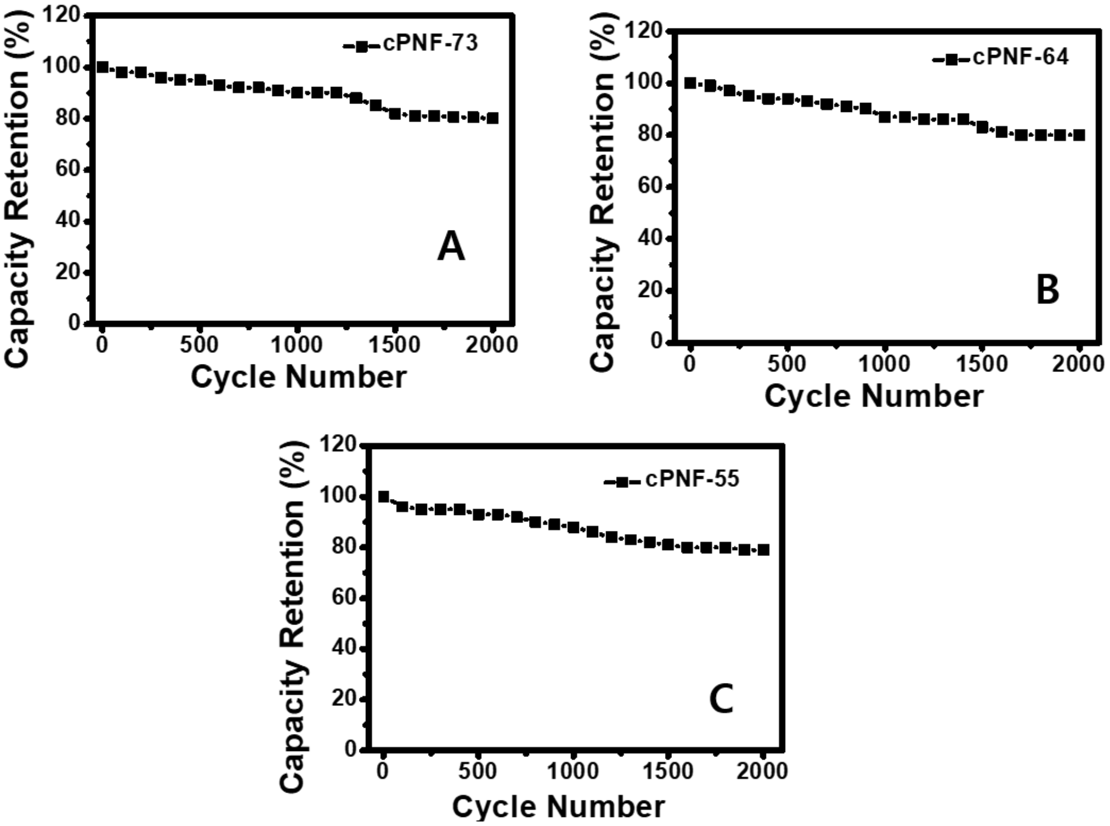

Figure S4. Cyclic stability of the fabricated cPNFs devices (A: cPNF-73, B: cPNF-64 and C: cPNF-55) at 2 A g^{-1}.

Table S1. Electrochemical performances of various hybrid supercapacitors

Electrodes	Operating Voltage	Specific current	Specific capacitance (F g^{-1})	Energy density (W h kg^{-1})	Power density (W kg^{-1})	Ref.
**Activated carbon nanofiber**	0.8	1	172	~ 4.3	~ 3	[1]
**Cornhusk carbon**	1.3	0.5	132	7.4	6.3	[2]
**rGO**	1	3	618	2.62	37.5	[3]
**CNT@Mn-MOF**	1	0.25	50.3	6.9	2.24	[4]
**N-doped porous nanofibers**	0.5	-	202	7.1	-	[5]
**cPAN-73**	1.45	1	428	1.74	0.38	This work

References

[1] Challagulla, N.V.; Vijayakumar, M.; Rohita, D.S.; Elsa, G.; Sankar, A.B.; Rao, T.N.; Karthik, M. Hierarchical activated carbon fibers as sustainable electrode and natural seawater as sustainable electrolyte for high performance supercapacitor. *Energy Technology* **2020** *8*, 2000417.

[2] Raj, C.J.; Manikandan, R.; Rajesh, M.; Sivakumar, P.; Jung, H.; Das, S.J.; Kim, B-C. Cornhusk mesoporous activated carbon electrodes and seawater electrolyte: The sustainable sources for assembling retainable supercapacitor module. *Journal of Power Sources* **2021** *490*, 229518.

[3] Madani, S.; Falamaki, C.; Alimadadi, H.; Aboutalebi, S.H. Binder-free reduced graphene oxide 3D structures based on ultra large graphene oxide sheets: High performance green micro-supercapacitor using NaCl electrolyte. *Journal of Energy Storage* **2019** *21*, 310-320.

[4] Zhang, Y.; Lin, B.; Sun, Y.; Zhang, X.; Yang, H.; Wang, J. Carbon nanotubes@metal-organic frameworks as Mn-based symmetrical supercapacitor electrodes for enhanced charge

storage. *RSC Advances* **2015**, *5*, 58100-58106.
[5] Chen, L-F.; Zhang, X-D.; Liang, H-W.; Kong, M.; Guan, Q-F.; Chen, P.; Wu, Z-Y.; Yu, S-H. Synthesis of nitrogen-doped porous carbon nanofibers as an efficient electrode material for supercapacitors. *ACS Nano* **2012** *89*, 7092-7102.
